# Molecular characterization and infectivity of a *Tomato leaf curl New Delhi virus *variant associated with newly emerging yellow mosaic disease of eggplant in India

**DOI:** 10.1186/1743-422X-8-305

**Published:** 2011-06-16

**Authors:** Dharmendra Pratap, Ashwin R Kashikar, Sunil K Mukherjee

**Affiliations:** 1 Plant Molecular Biology, International Centre for Genetic Engineering and Biotechnology, Aruna Asaf Ali Marg, New Delhi 110067, India; 2 Ankur Seeds Private Limited, Nagpur 440018, India

**Keywords:** Eggplant yellow mosaic disease, Begomovirus, *Tomato leaf curl New Delhi virus*, Betasatellite DNA, Agroinfiltration

## Abstract

**Background:**

Begomoviruses have emerged as serious problem for vegetable and fiber crops in the recent past, frequently in tropical and subtropical region of the world. The association of begomovirus with eggplant yellow mosaic disease is hitherto unknown apart from one report from Thailand. A survey in Nagpur, Central India, in 2009-2010 showed severe incidence of eggplant yellow mosaic disease. Here, we have identified and characterized a begomovirus responsible for the newly emerging yellow mosaic disease of eggplant in India.

**Results:**

The complete DNA-A and DNA-B genomic components of the causative virus were cloned and sequenced. Nucleotide sequence analysis of DNA-A showed that it shared highest 97.6% identity with *Tomato leaf curl New Delhi virus*-India[India:Udaipur:Okra:2007] and lowest 87.9% identity with *Tomato leaf curl New Delhi virus*-India[India:NewDelhi:Papaya:2005], while DNA-B showed highest 94.1% identity with ToLCNDV-IN[IN:UD:Ok:07] and lowest 76.2% identity with ToLCNDV-India[India:Lucknow]. Thus, it appears that this begomovirus is a variant of ubiquitous ToLCNDV and hence, we suggest the name ToLCNDV-India[India:Nagpur:Eggplant:2009] for this variant. The pathogenicity of ToLCNDV-IN[IN:Nag:Egg:09] isolate was confirmed by agroinfiltraion and dimeric clones of DNA-A and DNA-B induced characteristic yellow mosaic symptoms in eggplants and leaf curling in tomato plants.

**Conclusion:**

This is the first report of a ToLCNDV variant moving to a new agriculturally important host, eggplant and causing yellow mosaic disease. This is also a first experimental demonstration of Koch's postulate for a begomovirus associated with eggplant yellow mosaic disease.

## Background

Geminiviruses (family *Geminiviridae*) possess monopartite or bipartite circular, single-stranded DNA genomes encapsidated in geminate (18-22 nm diameters) particles. They infect a wide range of monocotyledonous and dicotyledonous plants but each member of the *Geminiviridae *family has its own limited host range. They are classified into four genera: *Mastrevirus, Curtovirus, Topocuvirus *and *Begomovirus*, on the basis of their genome organization, host range and insect vector [1,2]. A large number of important diseases are caused by begomoviruses, which comprises of more than 180 species [1]. Begomoviruses are transmitted by whitefly *Bemisia tabaci *(Gennadius) predominantly in the tropical and subtropical regions of the world and cause severe disease in dicot plants including tomato, pepper, cassava, beans, cotton and cucurbits [3-5].

The majority of the begomoviruses are bipartite and the genomic components are referred as DNA-A and DNA-B [1,6]. DNA-A encodes the replication-associated protein (Rep) which is essential for viral replication; the replication enhancer protein (REn); the transactivator protein (TrAP) that controls late gene expression and involved in RNAi suppression; and the coat protein (CP) for encapsidation and insect transmission. The DNA-B encodes the nuclear shuttle protein (NSP) and the movement protein (MP), both of which are vital in systemic spread and symptom expression. The two genomic components share a common region (CR) that contains motifs required for the control of gene expression and initiation of replication. CR has conserved reiterated motifs and a putative stem-loop structure containing the highly conserved nonanucleotide TAATATT↓AC, which is nicked by the Rep protein to initiate the rolling circle replication [7,8]. However, a few Old World begomoviruses such as *Tomato leaf curl virus *(ToLCV) and *Tomato yellow leaf curl virus *(TYLCV) are monopartite and have single genomic component, homologous to DNA-A of the bipartite begomoviruses. These monopartite viruses require only DNA-A to cause systemic infection [9-11].

In the recent past, novel satellite molecules called betasatellites (formerly known as DNA-β), were found to be associated with some monopartite begomoviruses such as *Cotton leaf curl Multan virus *(CLCuMV), which is responsible in the latest disease epidemic of cotton in Pakistan [12]. Betasatellites (~1.4 kb) have sequences unrelated to those of their helper begomoviruses but intriguingly, they depend on helper virus for replication, transmission and spread [13,14]. Betasatellites encode a single gene βC1, which is known to be a gene silencing suppressor [15]. Betasatellite is also known to play an important role in determining the host range of its associated begomovirus [14,16,17]. Some of the betasatellite molecules have relaxed trans-replication characteristics [18] and it has also been established that association of betasatellite increases disease severity and reduces the period between inoculation and appearance of symptoms [19]. It has also been shown that betasatellite can replace the movement function of DNA-B in case of bipartite begomoviruses [20].

Eggplant (*Solanum melongena *L.) commonly known as brinjal in India, is an economically important vegetable crop. The production of eggplant is severely affected by a number of plant viruses, particularly the RNA viruses [21]. Begomovirus association with eggplant is so far unknown except for one report from Thailand [1,22].

The natural occurrence of eggplant yellow mosaic disease (EYMD) was observed in a survey conducted in and around Nagpur region of Central India during 2009-2010 and the disease incidence was found to be around 60-65%. The disease prevails across the year and there is no significant seasonal variation about the severity of disease incidence. Infected plants showed severe yellow mosaic and mottling of leaves at later stage of infection. As the presence of whiteflies in the infected fields was noticed, we wanted to explore the possible association of begomoviruses with the EYMD in central India. We employed rolling circle amplification (RCA) technique to identify the infecting begomovirus. Our results revealed the clear association of a ToLCNDV isolate with EYMD in India.

Furthermore, we constructed dimeric agroinfectious clones from isolated genomic components and observed successful infectivity of these agro-clones in eggplants as well as in tomato plants. We have also recorded the period of disease appearance, symptom severity and accumulation of virus in the agroinfiltrated plants. We were also interested to investigate the degree of disease severity when the non-cognate betasatellite is infiltrated along with both the genomic components of the virus and for the purpose, the dimeric clone of non cognate betasatellite (CLCuMV) was used. To the best of our knowledge, this is the first record of a ToLCNDV variant moving to a new host, eggplant and causing EYMD in India and we have artificially simulated the new emerging disease thereby satisfying Koch's postulates.

## Methods

### Genomic DNA isolation

Different leaf samples of eggplant showing yellow mosaic symptoms were collected from various locations of Nagpur in Central India. From the fields surveyed, infected leaf samples from many different plants were collected and used for genomic DNA isolation. Total genomic DNA was isolated from infected samples by Cetyltrimethylammonium bromide (CTAB) method [23].

### Full length genomic amplification

Full length viral genomes (DNA-A and DNA-B) were amplified from the DNA isolated above using RCA [24] based TempliPhi™ DNA amplification kit (GE Healthcare). RCA reaction was performed as per manufacturer's instruction. The concatemers produced in the reaction were monomerized by restriction digestion with suitable restriction enzymes.

### Cloning and sequencing

Aliquots of 1 μl of the above RCA products were digested independently with various restriction enzymes: *Bam*HI, *Cla*I, *EcoRI, Hind*III, *Eco*RV, *Sac*I, and *Nco*I. Digested products were resolved on 1% agarose gel and the bands corresponding to ~ 2.7 kb genomes were purified using Hi Yield™Gel/PCR DNA Mini Kit. The 2.7 kb monomers (A or B) were cloned into the respective sites of pGreen0029 vector and henceforth, designated as pGreen-1.0A (*Sac*I) or pGreen-1.0B (*Xba*I). Monomeric full length clones were purified using HiYield™ Plasmid Mini Kit. Sequences of the recombinant clones were determined commercially by TCGA Company, India. We also investigated for any possible presence DNA-β satellite molecule in infected samples by RCA and also by Polymerase chain reaction (PCR) using the universal betasatellite primers, namely, Beta 01 and Beta 02 [25].

### Sequence analysis

Sequences of all the monomeric clones were assembled and analyzed using the software BIOEDIT version 7.0 programs [26]. Database searches with begomovirus sequences were carried out by NCBI-BLAST program (http://blast.ncbi.nlm.nih.gov). Nucleotide (nt) and amino acid (aa) sequence alignments were performed using CLUSTALW program using Mac Vector software (v11.1.2; MacVector Inc., USA). The phylogenetic tree was constructed using nucleotide sequences of complete DNA-A and DNA-B of ToLCNDV isolates and other selected begomovirus species reported from India and worldwide. Few other selected begomoviruses causing yellow mosaic disease in different plants were also taken into consideration so as to reveal the relationship of the virus isolate under study with its homologues. The phylogenetic tree was constructed with distance/neighbor-joining method with 1000 bootstrap replications and viewed with the help of MacVector suite program 10.5 (Mac Vector Inc, USA).

### Construction of agroinfectious clones and their infectivity

To check the infectivity of the above virus isolate, we used the high fidelity PCR based strategy for making dimeric clones of both DNA-A and DNA-B. For the purpose, we designed two different sets of abutting primers specific for amplifications of complete DNA-A and DNA-B genomic components of the virus isolate. For DNA-A, forward primer

5'-GAGCTCGTGCAGTTGTCCCCAT-3' and reverse primer

5'-AAGCTTCATAGGGGCTGTCGAAGTTGA-3' were synthesized commercially (IDT, USA). The nucleotides underlined represent the respective restriction sites. We incorporated one natural restriction site (*Sac*I) of the virus in the forward primer and one introduced restriction site (*Hind*III) in the reverse primer for the ease of cloning. The PCR conditions were as follows: initial denaturation at 94°C, 30 cycles at 94°C for 1 min, annealing for 1 min at 58°C, and extension for 3 min at 72°C, followed by a final extension of 10 min at 72°C. The PCR product was subsequently cloned in pGEM-T Easy vector (Promega) and the clones were confirmed by colony PCR, restriction digestion and sequencing. The 2.7 kb band, released by digestion with *Hind*III and *Sac*I from the recombinant pGEM-T Easy clone of DNA-A, was subsequently cloned at the same restriction sites of a binary vector pGreen 0029. This clone was named pG-A'. At the next step, the pGreen-1.0A plasmid DNA was digested with *Sac*I and the 2.7 viral kb DNA was recloned at the *Sac*I site of pG-A'. In this way, a complete head to tail DNA-A dimer was cloned in pGreen0029 (hereafter called as pGreen-2.0A). Insert integrity and orientation of the dimeric clones of DNA-A were confirmed by restriction digestion with *Dra*I, a unique cutter in the viral DNA sequence. Two bands corresponding to 7.3 and 2.7 kb dropped out following digestion with *Dra*I.

For construction of DNA-B dimeric clone, a similar strategy was adopted. The abutting primers were designed in a similar manner: forward primer 5'-TCTAGAACTCATTTGGTGTC-3' and reverse primer 5'-CTGCAGGAG AGAAACTGCAACTTCT-3'. The nucleotides underlined represent the respective restriction sites. We incorporated one natural restriction site (*Xba*I) in the forward primer and one introduced restriction site (*Pst*I) in the reverse primer. The PCR conditions were same as described above for DNA-A. The PCR products obtained were subsequently cloned in pGEM-T Easy vector (Promega) and the clones were confirmed by restriction digestion and sequencing. The 2.7 kb DNA released by digestion with *Xba*I and *Pst*I from pGEM-T Easy clone of DNA-B was subsequently cloned at the same restriction sites of binary vector pGreen0029. The recombinant clone was named pG-B'. The 2.7 kb viral DNA of pGreen-1.0B plasmid was subsequently mobilized at the *Xba*I site of pG-B' and the final dimeric clone was named pGreen-2.0B. Insert integrity and orientation of clones were confirmed by digestion with the internal cutter *Sca*I.

pGreen-2.0A and pGreen-2.0B plasmid DNAs were mobilized into competent *Agrobacterium tumefaciens *strain EHA105 with the helper plasmid p*Soup *by electroporation method using a Gene Pulser Apparatus[27]. *Agrobacterium *colonies were confirmed by colony PCR. The primers were designed from the flanking sequences of the inserts in the pGreen vector. Empty binary vector pGreen0029 in *Agrobacterium *was used as negative control for mock inoculation on control plants. To study the effects by betasatellite in disease severity, we also used dimeric agroinfectious clone of non-cognate CLCuMB betasatellite originated from northwestern India-CLCuMB[IN:ND1:03] [28].

*A. tumefaciens *cultures were incubated with shaking (200 rpm) at 28°C for 48 h (OD_600 _= 1) in yeast extract-Manitol (YEM) medium (pH 6.8) containing kanamycin (50 μg/ml), rifampicin (20 μg/ml) and chloramphenicol (34 μg/ml). Agrobacterium cells were harvested and resuspended in MES buffer [10 mM 2-(*N*-morpholino)ethanesulfonic acid **(**MES), 10 mM Magnesium chloride (MgCl_2_)] and used for agroinfiltration on test plants.

Eggplant (*Solanum melongena *cv.Pusa Purple Long) and tomato (*Solanum lycopersicum *cv. Pusa Ruby) plants were grown in vermiculite inside a temperature controlled glasshouse maintained at 25 ± 2°C and a 16/8 h light/dark cycle. 5-6 leaf stage eggplant and tomato plants were used for agroinfiltration. All the agroinfectious constructs, pGreen-2.0A, pGreen-2.0B and pBin-2.0β were used for infiltration in tomato and eggplants either individually or in various combinations with equimolar concentrations as described earlier [29]. Five plants each of eggplant and tomato plants were used for various combinations of agro-constructs and agroinfiltrated plants were grown in an insect-free glasshouse and the plants were maintained at 16/8 h light/dark periods for 30 weeks to observe the symptoms periodically.

### Analysis of viral DNA in inoculated plants

Total genomic DNA isolated from agroinfiltrated plants was used as a template for RCA. The templified products were digested with DNA-A specific single cutter restriction enzyme and the digested products were analyzed on agarose gel. Semiquantitative-PCR was also performed using ToLCNDV eggplant isolate DNA-A specific abutting primers to examine the relative viral accumulation efficiency of the virus in infiltrated test plants. The band intensities were quantified using Image J software. The quantified intensity values were normalized with respect to the respective actin controls which was taken as negative control. The value obtained for A+B was arbitrarily assigned as 100%. The sequence of actin primers and PCR conditions were similar as described earlier [30].

## Results

### Field detection of the viral disease and cloning of viral DNA

**Figure 1:**
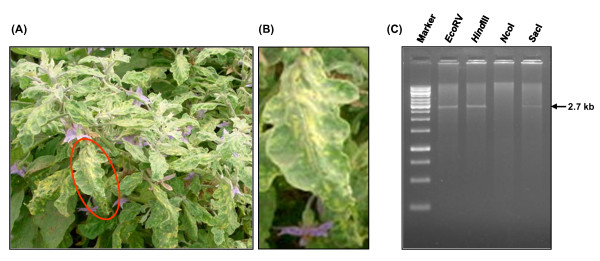
**(A) Naturally infected eggplants with yellow mosaic symptoms and mottling of leaves**. (B) A magnified image of a part of the diseased leaf [encircled in red (A)]. (C) The profile of restriction digestions of the RCA products of DNA derived from the leaves of naturally infected plants. The DNA marker is shown at the extreme left of the figure. The enzymes used for digestion are indicated at the top of the figure.

Eggplants showing severe yellow mosaic symptoms were observed in fields during a survey carried out in 2009-2010 in central India (Figure 1A, B). To characterize the presence of begomovirus in the infected plants, total DNA was isolated from the leaf samples of infected plants as well as from healthy eggplants (as negative control). RCA was performed with the isolated DNA and the RCA products were digested with different restriction enzymes. The results presented in Figure 1C clearly demonstrate the presence of ~2.7 kb product in case of infected samples when digested with *Hind*III, *Eco*RI, *Bam*H1, *Sac*I and *Eco*RV, while no such band appeared on digestion with *Nco*I. The appearance of 2.7 kb amplified DNA suggested the presence of a begomovirus in the infected leaves. In healthy leaf samples no band corresponding to 2.7 kb was observed, as expected. The 2.7 kb fragments obtained from digestion with above restriction enzymes were directly cloned in pGreen0029 at their respective sites and sequenced. The DNA was extracted separately from the leaves of 15 different infected plants of same neighborhood and subjected to similar treatments of RCA followed by restriction digestion. In every case, similar results were obtained and the amplified and digested DNAs were cloned and sequenced. About twenty clones from the leaf DNA of each infected plant were sequenced.

### Viral DNA sequence analysis

The end sequences (about 700 bases in total), obtained from each of the positive samples, showed the presence of a bipartite (DNA-A and DNA-B) begomovirus. All DNA-A clones were found to be more than 99% identical amongst themselves and all the DNA-B clones were also about 97% identical amongst themselves. Hence, for subsequent studies, only a few clones of each type, i.e., having either DNA-A or DNA-B, were chosen for further characterization of the virus. To sequence the remaining portion of DNA-A and DNA-B, primer walking was carried out by designing additional internal primers. Three clones of each of DNA-A and DNA-B were sequenced completely and one such clone of each DNA-A and DNA-B was used for characterization of the full genome. The restriction enzymes *Sac*I and *Eco*RV for DNA-A and *Hind*III and *Xba*I for DNA-B were found to be unique cutters. The complete DNA-A and DNA-B sequences are available in GenBank with the accession numbers HQ264185 and HQ264186 respectively. The DNA-A and DNA-B components are comprised of 2741 nt and 2698 nt respectively and their genome organizations are identical to those of the previously characterized bipartite begomoviruses. DNA-A contains six open reading frames (ORFs) of which two ORFs AV1 and AV2 are in the virion sense and the other four ORFs, i.e., AC1, AC2, AC3 and AC4, are in complementary sense. These ORFs are interspersed by 276 nt long intergenic region (IR) located between the ORFs AC1 and AV2. The DNA-B component contains two ORFs designated as BVI on the sense strand and BC1 on the complementary strand. The DNA-A and DNA-B genomes did not exhibit any sequence similarity, except for approximately 160 nt common region (CR) which showed ~96.8% sequence identity with each other. In the CR, a 34 bp potential stem-loop forming region (5'-GCGGCCATTCGTATAATATTACCGAATGGCCGCG-3') was identified which includes the conserved nonanucleotide sequence TAATATTAC present within the replication origin of almost all geminiviruses.

The nucleotide sequence comparison of complete DNA-A of the virus isolate with the selected strains of TOLCNDV, TYLCV and other begomoviruses reported from India and worldwide (Table 1) showed highest identity (97.6%) with ToLCNDV-IN[IN: UD: Ok: 07] and lowest (87.9%) with ToLCNDV-IN[IN:ND:Pap:05] (Table 2). We thus suggest the name of the variant as ToLCNDV-India[India:Nagpur:Eggplant:2009] in accordance with the latest International Committee on Taxonomy of Viruses (ICTV) guidelines for species demarcation in the genus [1].

**Table 1:** Name, acronym and GenBank accession numbers of the selected begomovirus genome sequences used for study.

Begomoviruses	DNA-A*	DNA-B*	Acronym
*Tomato leaf curl New Delhi virus*- India [India: New Delhi:2005]	DQ169056	DQ169057	ToLCNDV-IN[IN:ND:05]

*Tomato leaf curl New Delhi virus-*India[India:Udaipur:Okra:2007]	EF035482	EF043394	ToLCNDV-IN-[IN:UD:Ok:07]

*Tomato leaf curl New Delhi virus-*Bangladesh	EF450316	NA	ToLCNDV-BD

*Tomato leaf curl New Delhi virus -*OM-Taiwan	GU180095	GU180096	ToLCNDV-[OM: Tai]

*Tomato leaf curl New Delhi virus-*Pakistan	EF620534	EF620535	ToLCNDV-Pak

*Tomato leaf curl New Delhi virus *- India-186b	GQ865546	NA	ToLCNDV-IN-186b

*Tomato leaf curl New Delhi virus *[India:Bangalore:OY135:2005]	GU112084	GU112085	ToLCNDV-IN[IN: Ban:05]

*Tomato leaf curl New Delhi virus *- India[Pakistan:Solanum nigrum:1997]	AJ620187	AJ620188	ToLCNDV-IN[PK: Sn: 97]

*Tomato leaf curl New Delhi virus*- India[India:New Delhi:Severe:1992]	U15015	U15017	ToLCNDV-IN[IN: ND: Svr:92]

*Tomato leaf curl New Delhi virus*-India [Bangladesh:Jessore: Severe:2005]	AJ875157	AJ875158	ToLCNDV-IN[BG:Jes:Svr:05]

*Tomato leaf curl New Delhi virus*-India [India: Lucknow]	Y16421	X89653	ToLCNDV-IN[IN: Luck]

*Tomato leaf curl New Delhi virus *-India [India: Happur: Potato: 2005]	EF043230	EF043233	ToLCNDV-IN[IN:Hap:Pot:05]

*Tomato leaf curl New Delhi virus *-India[India: Meerut: Potato: 2005]	EF043231	EF043232	ToLCNDV-IN[IN: Mer: Pot: 05]

*Tomato leaf curl New Delhi virus*-Thailand[Cucumber: Thailand]	AB330079	AB330080	ToLCNDV-TH[Cuc:Thai]

*Tomato leaf curl New Delhi virus*-India [India: New Delhi: Mild :1992]	U15016	NA	ToLCNDV-IN[IN: ND: Mld:92]

*Tomato leaf curl New Delhi virus*-Thailand [Luffa:Thailand]	AF102276	NA	ToLCNDV-TH[TH: Luf]

*Tomato leaf curl New Delhi virus*-India [India: New Delhi:Papaya:2005]	DQ989325	NA	ToLCNDV-IN[IN:ND:Pap:05]

*Tomato leaf curl New Delhi virus *[India:Aurangabad:OY164A:2006]	GU112088	GU112089	ToLCNDV-IN[IN: Aug: 06]

***Tomato leaf curl New Delhi virus *[India:Nagpur:Eggplant:2009]**	HQ264185	HQ264186	**ToLCNDV-IN[IN:Nag:Egg:09]**

*Tomato leaf curl Palampur virus*-India	AM884015	AM992534	ToLCPMV

*Squash leaf curl China virus *[Pumpkin :Coimbatore]	AY184487	AY184488	SLCCV-IN[Pump:Coi]

*Tomato yellow leaf curl Kanchanaburi virus*[Thailand Kanchanaburi 2: Eggplant: 2001]	AF511530	AF511528	TYLCKaV-TH[TH:Kan2:Egg:01]

*Tomato yellow leaf curl Kanchanaburi virus*[Vietnam: Binhudong: Eggplant:2005]	DQ641702	NC_005811	TYLCKaV-TH[Vn:Bin:Egg:05]

The ToLCNDV variant under study is written in bold.

**Table 2:** Percent identities (nucleotide^#^) of ToLCNDV-IN[IN:Nag:Egg:09] with DNA-A and DNA-B of selected begomoviruses reported worldwide.

Virus-Acronym	DNA-A	DNA-B
ToLCNDV-IN[IN:UD:Ok:07]	97.6	94.1
ToLCNDV-IN-186b	97.0	NA
ToLCNDV-IN[IN: Ban:05]	96.3	86.4
ToLCNDV-IN[IN: Aug: 06]	95.6	88.3
ToLCNDV-IN[IN:ND:05]	94.5	86.4
ToLCNDV-IN[PK: Sn: 97]	94.5	82.0
ToLCNDV-IN[IN: ND: Svr:92]	94.3	86.4
ToLCNDV-IN[BG:Jes:Svr:05]	94.3	86.4
ToLCNDV-IN[IN: Luck]	94.0	76.2
ToLCNDV-IN[IN: Mer: Pot: 05]	93.7	86.3
ToLCNDV-Pak	93.2	90.9
ToLCNDV-TH[Cuc:Thai]	92.7	81.4
ToLCNDV-IN[IN: ND: Mld:92]	92.7	NA
ToLCNDV-[OM: Tai]	92.1	80.6
ToLCNDV-IN[IN:Hap:Pot:05]	91.4	86.7
ToLCNDV-TH[TH: Luf]	91.1	NA
ToLCNDV-BD	88.8	NA
ToLCNDV-IN[IN:ND:Pap:05]	87.9	NA
SLCCV-IN[Pump:Coi]	86.8	63.3
TolCPMV	85.3	68.1
TYLCKaV-TH[Vn:Bin:Egg:05]	67.3	39.0
TYLCKaV-TH[TH:Kan2:Egg:01]	66.9	39.0

^# ^Information about the sequences used for comparison is provided in Table1

NA-Sequence not available

The nucleotide sequence analysis of DNA-B of the ToLCNDV isolate under study with selected DNA-B sequences of begomovirus in NCBI database showed that it has highest sequence identity (94.1%) with ToLCNDV-IN[IN:UD:Ok:07] and lowest with ToLCNDV-IN[IN: Luck] (Table 2).

**Figure 2:**
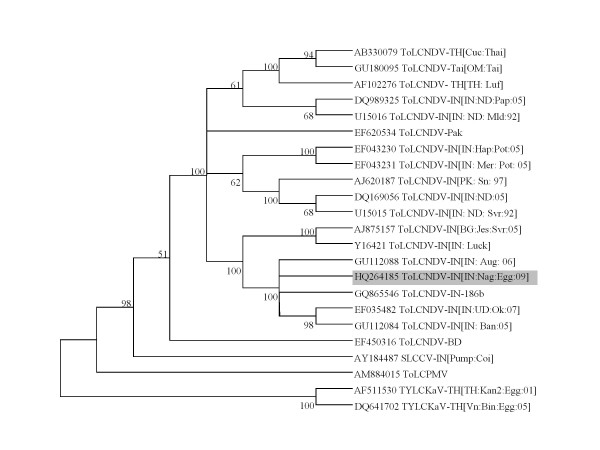
**Phylogenetic tree of complete DNA-A of ToLCNDV-IN[IN: Nag: Egg: 09]**. The phylogenetic tree was constructed with distance/neighbour-joining method with 1000 bootstrap replications and viewed with the help of MacVector suite program 10.5 (Mac Vector Inc, USA).

**Figure 3:**
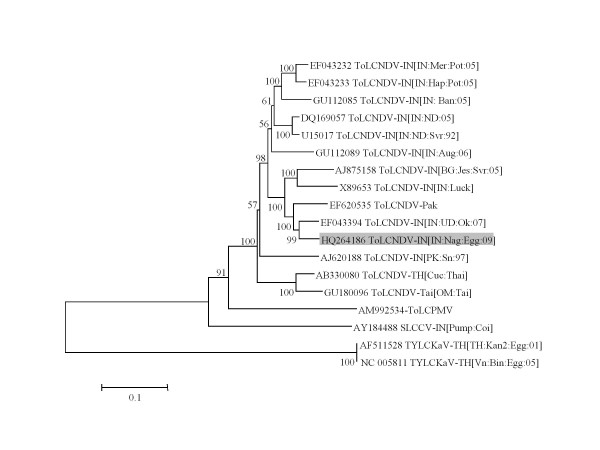
**Phylogenetic tree of complete DNA-B of ToLCNDV-IN[IN:Nag:Egg:09]**. The phylogenetic tree was constructed with distance/neighbour-joining method with 1000 bootstrap replications and viewed with the help of MacVector suite program 10.5 (Mac Vector Inc, USA).

The phylogenetic tree analysis on the basis of nucleotide sequence of DNA-A genome of the ToLCNDV-IN[IN:Nag:Egg:09] with the DNA-A sequences of other selected begomoviruses revealed that the ToLCNDV-IN[IN:Nag:Egg:09] isolate has close relationship with the strains of ToLCNDV. In contrast, DNA-A nucleotide sequences of begomovirus species associated with EYMD in Thailand, showed divergent relationship with ToLCNDV-IN[IN: Nag: Egg: 09] isolate (Figure 2). Phylogenetic analysis of the DNA-B genome also showed that ToLCNDV-IN[IN:Nag:Egg:09] isolate has close relationship with ToLCNDV strains (Figure 3).

### Infectivity of the cloned genomic components

**Figure 4:**
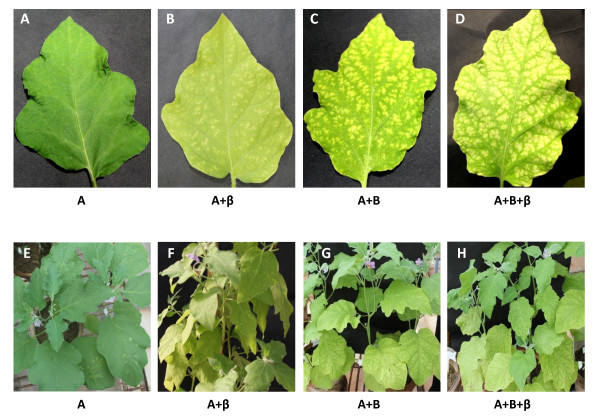
**The disease was induced by ToLCNDV-IN[IN:Nag:Egg:09] in eggplants that were inoculated with different constructs as labeled**. The agro-inoculated plants are shown at the bottom panel and the corresponding representative leaves are shown in the top panel in the magnified form to reveal the patterns of disease symptoms at around 90 dpi.

**Figure 5:**
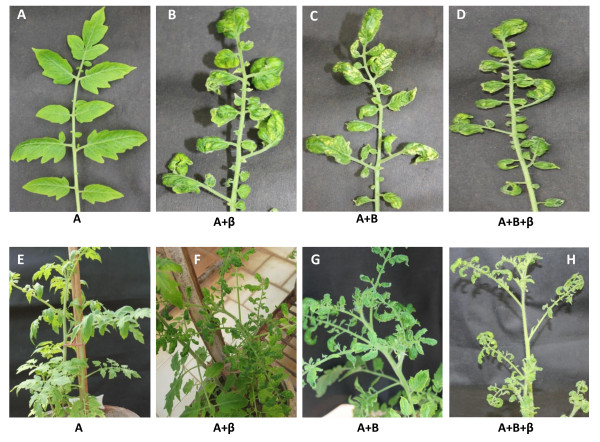
**Plants were inoculated with different constructs as labeled**. Rest of the condition is same as described in Figure 4.

To assess the infectivity of the present ToLCNDV isolate, agroinfectious constructs of both the genomes were introduced into the leaves of tomato as well as eggplant using the agro-infiltration technique. Agroinfiltration of various constructs were carried out either individually or in various combinations and the consequent disease expressions are summarized in Table 3. When both the genomic components (DNA-A and DNA-B) were co-infiltrated, typical yellow mosaic symptoms at 60 days post inoculation (dpi) was observed in eggplants (5/5) (Figure 4, panels C and G) and the same constructs evoked typical downward leaf curling in tomato plants (5/5) at 45 dpi (Figure 5, panels C and G). The symptoms persisted even at 150 and 180 dpi in eggplants and tomato plants respectively.

**Table 3:** Infectivity and symptom induced by ToLCNDV-IN[IN:Nag:Egg:09] with or without non-cognate betasatellite CLCuMB-[IN:ND1:03] and the number of symptomatic plants as confirmed by RCA.

Host/Inoculated dimeric agro-constructs	Symptomatic plants/Inoculated plants	Types of symptoms (90dpi)
**Eggplant**		
DNA-2A	0/5	No Symptoms
DNA-2A+DNA-2B	5/5	Yellow mosaic
DNA-2A+ DNA-2β	3/5	Mild yellow mosaic
DNA-2A+ DNA-2B + 2β	5/5	Severe yellow mosaic
DNA-2B	0/5	No Symptoms
DNA-2β	0/5	No Symptoms
DNA-2B+ CLCuMV-DNA-2β	0/5	No Symptoms
**Tomato**		
DNA-2A	0/5	No Symptoms
DNA-2A+ DNA-2B	5/5	Leaf curling
DNA-2A+ DNA-2β	4/5	Mild leaf curling
DNA-2A+ DNA-2B + DNA-2β	5/5	Severe leaf curling with occasionally yellow mosaic
DNA-2B	0/5	No Symptoms
DNA2β	0/5	No Symptoms
2B+ 2β	0/5	No Symptoms

To investigate the possible role of non-cognate DNA-β satellite in the disease severity, we co-infiltrated the dimeric clone of betasatellite, CLCuMB[IN:ND1:03] along with both the genome-components of ToLCNDV-IN[IN:Nag:Egg:09] on the test plants (eggplant and tomato). The eggplants exhibited severe yellow mosaic symptoms at 45 dpi (Figure 4, panels D and H) while severe downward leaf curling was observed in tomato at 30 dpi (Figure 5, panels D and H).

The DNA-A genomic component failed to develop disease either in eggplant or tomato when infiltrated alone (Figure 4, panels A and E; Figure 5, panels A and E). In addition, we were also interested to examine that the DNA-B component of the ToLCNDV-IN[IN:Nag:Egg:09] isolate could be replaced by non-cognate CLCuMV DNA-β for systemic disease development. As anticipated, co-infiltration of DNA-A and DNA-β resulted in successful systemic disease development. The symptoms were mild mosaic in eggplant (2/5) (Figure 4, panels B and F) and leaf curling in tomato plants (3/5) (Figure 5, panels B and F). Thus, non cognate CLCuMV DNA-β substituted DNA-B of ToLCNDV-IN[IN:Nag:Egg:09] for systemic viral movement in both eggplant and tomato plants, albeit in a reduced manner. However, the disease expression period was prolonged compared to when the B-component was administered with DNA-A. The symptom development periods were 90 and 80 dpi for eggplant and tomato respectively.

However, the severity of the symptoms was most pronounced when all of the three components, i.e., DNA-A/DNA-B/DNA-β, were co-transferred in plants. Figure 4 shows the severity of yellow mosaic disease in eggplants and the leaves curled with progression of the disease (panels D and H). The disease expression time also reduced to 45 days. Similar was the case with tomato and the curled leaves showed mosaic symptoms eventually due to virus infection (Figure 5, panels D and H). The disease also expressed early, i.e., at about 30 dpi in tomato plants.

As expected, neither empty pGreen vector (negative control) nor DNA-B and DNA-β construct caused any symptoms in test plants even at 180 dpi and no noticeable phenotypic change in eggplant and tomato plants were observed. In contrast, plants infiltrated with the agroinfectious constructs were always stunted in growth.

### The level of viral DNA accumulation in the diseased plants

In order to know the level of viral DNA accumulation, total DNA from systemic and symptomatic leaves of agroinfiltrated eggplant and tomato plants was extracted and subjected to semi-qPCR and RCA. RCA products of high molecular weight were digested with *Eco*RV, unique site in the DNA-A of ToLCNDV-IN[IN:Nag:Egg:09]. The 2.7 kb band was observed only in plants agro infiltrated with either both the genomic components or DNA-A with non cognate DNA-β. However, no such band was observed in plants infiltrated with only DNA-A (data not shown).

**Figure 6:**
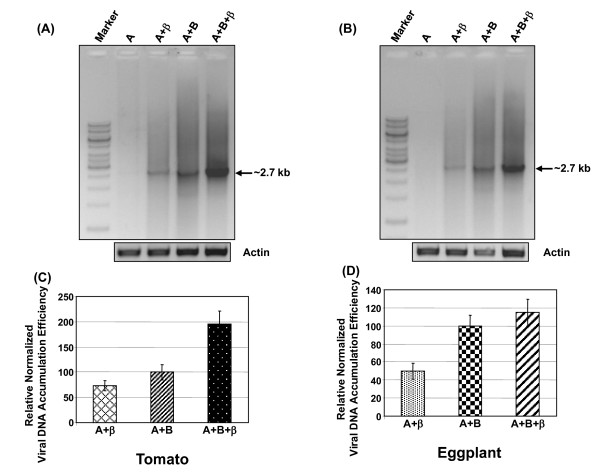
**The plants (Tomato and Eggplant) were agro-infiltrated with various constructs as shown on the top of the figures, and the level of viral DNA accumulation was checked by semi-qPCR at 90 dpi using DNA-A specific abutting primers ToLCNDV-IN[IN:Nag:Egg: 09]**. The amplification products (~2.7 kb) were separated on 1% agarose gel and are presented in panels (a) and (b) for tomato and eggplant respectively. The band intensities were quantified and the normalized values with respect to corresponding loading controls (ACTIN) are plotted as bar graphs in panels (c) and (d) respectively. The standard deviations shown are based on three independent experiments.

The semi-qPCR was carried out to know the level of viral DNA accumulation in plants infiltrated with various constructs. The results revealed that the viral DNA accumulation in tomato plants was highest when the plants were infiltrated with all the three components, namely DNA-A, DNA-B and non cognate DNA-β. The accumulation of viral DNA was lowest when the DNA-A and DNA-β was used for co-infiltration (Figure 6A, C). Similar results were also obtained with eggplants (Figure 6B, D). However, the effects of non cognate β DNA was less pronounced in eggplants compared tomato.

The measured intensities of the 2.7 kb bands were normalized with respect to the corresponding actin controls. The average normalized intensity values (based on three independent studies) are presented as bar graphs in Figure 6C, D. Our data reflect that the accumulation of viral DNA increased to ~95% on introduction of non cognate DNA β in tomato while there was a modest increase of ~14% in eggplant. The accumulation of viral DNA was further reduced up to ~27% when DNA-B was substituted by non cognate DNA-β in tomato plants whereas the same was ~50% in eggplant (Figure 6C, D).

## Discussion

Eggplant is one of the preferred hosts for rearing of whiteflies and was known to be immune for begomovirus [31] till the recent past. However, begomovirus causing EYMD in Thailand have been identified [1,22] although, the etiology of the disease was not satisfied by Koch's postulates. Prominent yellow mosaic symptoms were observed on eggplants in fields during 2009-2010 in Nagpur, India. Virus like symptoms and infestation of whiteflies prompted us to investigate the possible association of begomovirus with the newly emerging disease of eggplant. Our results confirmed the association of a begomovirus with EYMD. Based on high nucleotide sequence identity (97.6% for DNA-A and 94.1% for DNA-B) with the strains of ToLCNDV, phylogenetic analysis and the demarcation criteria in species demarcation[1], the begomovirus isolated from diseased eggplant is considered as a variant of ToLCNDV and we suggest the name as ToLCNDV-IN[IN:Nag:Egg:09].

ToLCNDV is an economically important pathogen and found to be associated with various crop plants in India, Pakistan, Thailand and Bangladesh [1]. ToLCNDV has been reported for the first time in India from tomato and it required both DNA-A and DNA-B for symptom development [2]. Later on, it has been reported from various crops and weeds such as, chili [32], cucurbits [33], potato [34], papaya[35], bittergourd [36], cotton (GenBank Accession number, EF063145) and *Solanum nigrum *(GenBank Acc. No. AJ620187). As already known, virus isolated from a plant may or may not be the cause of disease unless it satisfies the Koch's postulates and despite a large host range and geographical distribution of ToLCNDV, very few cloned isolates have been shown to be infectious to either experimental plants or the hosts from which they were isolated.

Therefore, to satisfy the Koch's postulates, the infectivity of ToLCNDV-IN[IN:Nag:Egg:09] was established by infiltrating the agroinfectious clones in eggplant. The agroinfiltration in eggplants resulted in the disease symptoms that are similar to those occurred in the virus infected eggplant in fields. Hence, we fulfilled the Koch's postulates and showed for the first time that ToLCNDV-IN[IN:Nag:Egg:09] is responsible for the newly emerging EYMD in central India.

Interestingly, ToLCNDV-IN[IN:Nag:Egg:09] gave the typical leaf curling symptoms, not the yellow mosaic in tomato plants. Thus, these results also indicated that the nature of symptoms and disease is largely a host-driven process in this case. The successful virus infection is dependent on productive interactions between viral and host factors at each stage of the infection process and host-adaptation have a different basis in different plants [37,38].

On agroinfiltration with empty vector (taken as negative control) or only DNA-A of ToLCNDV-IN[IN:Nag:Egg:09], no symptoms were recorded even by 180 dpi. This showed that DNA-A alone of ToLCNDV-IN[IN:Nag:Egg:09] was neither able to sustain nor produce systemic infection in the host plants. This phenomenon is perhaps true for most of the geminiviruses with the bipartite genomes [2,15].

The association of a DNA-β molecule with the field infection of ToLCNDV-IN[IN:Nag: Egg:09] could not be detected by either PCR or RCR method. Nevertheless, we still suspect that in succeeding time, betasatellite associated monopartite begomovirus may be identified with EYMD in southern India. Consequently, we used the non-cognate CLCuMB[IN: ND1:03] molecule to examine its role in degree of virus infection. When CLCuMB[IN: ND1:03] was co-infiltrated with DNA-A of ToLCNDV-IN[IN:Nag:Egg:09], produced mild leaf curling in tomato plants. This result suggested that CLCuMV DNA-β can successfully substitute the DNA-B for systemic movement of ToLCNDV-IN[IN:Nag:Egg:09] and symptom development. These results are in conformity with the earlier findings that DNA-β can substitute DNA-B for systemic movement [20]. However, the symptoms took longer time to develop and they were mild comparative to that when both the genomic components (DNA-A and DNA-B) were present. It indicated that DNA-β can substitute DNA-B up to certain extent but not completely. These results are also in accordance with the previous findings that, DNA-B plays the major role in symptom production and viral pathogenicity in bipartite begomovirus [39].

The DNA-β satellite molecules are also known for intensification of disease symptoms in a host dependent manner [27,40]. Co-infiltration of DNA-A and DNA-B of ToLCNDV-IN [IN:Nag:Egg:09] with DNA-β of CLCuMV resulted in enhanced severity of disease in both eggplant and tomato. The appearance of disease symptoms also took lesser time compared to that required by both DNA-A and DNA-B. Thus, our results promoted the role of DNA-β in increasing the disease severity[41].

The symptomatic variation with agroinfiltrated test plants which were infiltrated with various combinations of constructs were corroborated with molecular findings as the relative virus accumulation of virus increased to about ~95% and ~14% in tomato and eggplant respectively when DNA-β was infiltrated along with both DNA-A and DNA-B. The value reduced to ~27% and ~50% respectively in tomato and eggplant when DNA-B was replaced with DNA-β, where the value obtained for A+B was arbitrarily assigned as 100%. Furthermore, these results support the notion that DNA-β increases the disease severity by increasing the accumulation of the helper virus. The variation observed in tomato and eggplant may be due to the fact that DNA-β increased the symptoms severity in a host dependent manner [14,42].

Thus, we have identified and characterized the virus associated with EYMD as a variant of ubiquitous TOLCNDV, but presence of new begomoviruses species with the eggplant cultivation in India cannot be negated as different species of monopartite and bipartite begomoviruses exist across India and among them many monopartite begomoviruses with betasatellite molecules are already known to be prevalent in southern India [43]. The diversity of begomovirus may lead to adaptation of eggplants as a new host and as a result the eggplants in other part of India might be under severe threat. Therefore, some practical intervention measures, such as enforcement of quarantine regulations in the trade of live plant materials and development of resistant plants are urgently needed to curb the viral threat.

## Conclusion

Taken together, on the basis of molecular characterization and infectivity test, we can conclude that ToLCNDV-IN[IN:Nag:Egg:09] is a newly emerging variant of ToLCNDV moving to a new economically important host, eggplant and subsequently posing severe constraint on eggplant production in India. This is also a first experimental demonstration of Koch's postulate for a begomovirus associated with eggplant yellow mosaic disease.

## Competing interests

The authors declare that they have no competing interests.

## Authors' contributions

DP carried out the entire experiments. ARK carried out the survey and collected the infected samples. SKM conceived of the study, DP and SKM participated in designing the experiments. DP and SKM prepared the manuscript. All authors read and approved the final manuscript.
